# Pre-emptive detection and evolution of relapse in acute myeloid leukemia by flow cytometric measurable residual disease surveillance

**DOI:** 10.1038/s41375-024-02300-z

**Published:** 2024-06-18

**Authors:** Nicholas McCarthy, Gege Gui, Florent Dumezy, Christophe Roumier, Georgia Andrew, Sarah Green, Madeleine Jenkins, Alexandra Adams, Naeem Khan, Charles Craddock, Christopher S. Hourigan, Adriana Plesa, Sylvie Freeman

**Affiliations:** 1 Institute of Immunology and Immunotherapy, University of Birmingham, Birmingham, UK; 2 Laboratory of Myeloid Malignancies, Hematology Branch, National Heart, Lung, and Blood Institute, National Institutes of Health, Bethesda, MD USA; 3 Johns Hopkins Bloomberg School of Public Health, Baltimore, MD USA; 4 Laboratory of Hematology, Lille University Hospital, Lille, France; 5 Imperial College, London, UK; 6 University of Edinburgh, Edinburgh, UK; 7 Clinical Trials Unit, University of Warwick, Coventry, UK; 8 Lyon University Hospital, CHU-HCL, Lyon Sud, Pierre Benite, France

**Keywords:** Leukaemia, Risk factors, Medical research

## Abstract

Measurable residual disease (MRD) surveillance in acute myeloid leukemia (AML) may identify patients destined for relapse and thus provide the option of pre-emptive therapy to improve their outcome. Whilst flow cytometric MRD (Flow-MRD) can be applied to high-risk AML/ myelodysplasia patients, its diagnostic performance for detecting impending relapse is unknown. We evaluated this in a cohort comprising 136 true positives (bone marrows preceding relapse by a median of 2.45 months) and 155 true negatives (bone marrows during sustained remission). At an optimal Flow-MRD threshold of 0.040%, clinical sensitivity and specificity for relapse was 74% and 87% respectively (51% and 98% for Flow-MRD ≥ 0.1%) by ‘different-from-normal’ analysis. Median relapse kinetics were 0.78 log_10_/month but significantly higher at 0.92 log_10_/month for *FLT3*-mutated AML. Computational (unsupervised) Flow-MRD (C-Flow-MRD) generated optimal MRD thresholds of 0.036% and 0.082% with equivalent clinical sensitivity to standard analysis. C-Flow-MRD-identified aberrancies in HLADRlow or CD34+CD38low (LSC-type) subpopulations contributed the greatest clinical accuracy (56% sensitivity, 90% specificity) and notably, by longitudinal profiling expanded rapidly within blasts in > 40% of 86 paired MRD and relapse samples. In conclusion, flow MRD surveillance can detect MRD relapse in high risk AML and its evaluation may be enhanced by computational analysis.

## Introduction

Detection of impending relapse in acute leukemias allows selection of patients for pre-emptive therapies that may avoid ultimate treatment failure but also the challenges of cytoreduction and morbidities following hematological relapse. In acute myeloid leukemia, the evidence that sequential MRD testing can provide an early warning of relapse comes predominantly from studies applying real-time quantitative polymerase chain reaction (RT-qPCR) assays [1, 2]. Most of this data is for common PCR MRD leukemia targets *PML-RARA*, *CBFB-MYH11*, *RUNX1-RUNX1T1*, and *NPM1* mutations although the relapse kinetics of other PCR targets, namely rarer rearrangements and overexpressed WTI have also been examined [3–5]. Based on the findings of these studies, definitions of treatment failure now include molecular MRD relapse (increase of MRD copy numbers ≥ 1 log_10_ or conversion from MRD negativity to MRD positivity, confirmed in a second sample) and RT-qPCR surveillance for MRD relapse after treatment (3 monthly if bone marrow sampling) is recommended for CBF and *NPM1* mutated AML [2]. The term MRD relapse now also incorporates conversion from MRD negativity to positivity detected by other methods including flow cytometry and is thus applicable across AML subtypes [2]. There remain however unresolved issues that limit the information from sequential MRD results and therefore the clinical benefit and opportunities from MRD detection of impending relapse. These in part arise from the challenges of determining clinical specificity and sensitivity of a result to predict relapse when applying the recommended monitoring schedule. Conversion from MRD negativity to low level MRD positivity may have low predictive accuracy for relapse (low specificity), even when the sample MRD target is a high risk leukemic transcript such as *DEK-NUP214* [5]. Although a second consecutive sample showing rising MRD levels can confirm MRD relapse, this risks reducing the interval for treatment decisions in the context of often unpredictable relapse kinetics. Furthermore, the majority of hematological relapses may not be identified by a preceding MRD positive test (low clinical sensitivity) despite using the recommended 3 monthly bone marrow monitoring schedules with established highly sensitive qPCR assays such as applied to CBF AML patients [6]. Additionally, as the use of flow cytometry or NGS for surveillance of impending relapse remains exploratory in AML [2, 7], there is an unmet need to extend MRD relapse detection to all AML patients. Moreover, parallel molecular and flow cytometric serial surveillance may be necessary for some patients such as when there is a risk of clonal evolution [8] including from treatment escape with loss of the molecular MRD target [9]. Although flow cytometric MRD has the advantage of rapid turn-around time in addition to a sensitivity of at least 10^-4^, the current dependence on manual analysis of bidimensional plots can lead to inconsistent quantitation according to expertise [10] and also limit the potential of deeper-immunophenotyping for further optimization.

We recently demonstrated the prognostic value of flow cytometric MRD monitoring performed during the first year following allogeneic stem cell transplantation [11]. This study now evaluates the clinical sensitivity and specificity of flow cytometric bone marrow MRD surveillance for early relapse prediction. We compare standard MRD detection with a newly developed computational analysis approach, investigating assay performance and aberrant immunophenotypic populations that are most specific for imminent relapse.

## Methods

### Samples and patients

Patient samples were from AML patients > 18 years old followed for flow cytometric measurable residual disease (MRD) detection using standard published methods [2, 12–15] by a single reference laboratory (Birmingham, UK) from April 2015 to September 2022. MRD bone marrow monitoring was conducted after completion of chemotherapy or post allogeneic hematopoietic stem cell transplantation (HSCT) in patients who had achieved a complete remission (CR) or CR with incomplete hematologic recovery (CRi). MRD detection analyses were conducted with informed patient consent in accordance with the Declaration of Helsinki and subject to appropriate ethics committee approval (NHS Health Research Authority REC reference 20/NW/0286).

MRD bone marrow samples preceding a hematological relapse (excluding APML patients) were retrospectively identified and included when a paired relapse sample had been received up to 4 months after the MRD sample with the diagnosis of relapse confirmed as at the timepoint of the relapse sample (Supplementary Fig. 1).

AML MRD monitoring control samples were defined as 1) post-treatment PCR negative bone marrows from patients with acute promyelocytic leukemia (APML) maintaining molecular remission (test control cohort) and 2) post treatment / HSCT bone marrows from non-favourable risk AML patients in continuous remission >6 months after MRD sample without a treatment intervention (validation control cohort). (Supplementary Fig. 1).

Additionally, pre-transplant bone marrow files from 156 patients entered into the FIGARO trial with previously reported standard flow cytometric MRD results [14] were included for clinical validation of computational analysis.

### Flow cytometric MRD testing and analysis

MRD was assessed by flow cytometry as previously described in a central reference laboratory) [13, 14] Details on sample logistics, processing, panel and analysis strategy are provided in the Supplementary Methods. AML MRD panel was 7 colour until 2017 [13] and 8 colour thereafter [15]. Routine flow cytometric MRD analysis was performed using a standardized manual gating strategy that screened blasts for different-from-normal (DfN) aberrant immunophenotypes that were established as useful and frequently observed leukemic aberrant immunophenotypes (LAIPs) and also for any previously identified baseline LAIPs when available. Samples were reported as MRD-negative if no baseline and/or different-from-normal LAIP cells could be quantitated above assay threshold (of 0.05%).

To confirm the results of standard analysis, and exclude variability arising from subjective interpretation, flow cytometry standard files from MRD testing were analysed using a computational approach (C-Flow MRD), updated following previous clinical evaluation [11, 14]. Blast cells (CD117 + /CD34 + ) from test samples were clustered together with a 40-50 control BM reference set using the FlowSOM clustering algorithm. Automated decision tree analysis was then applied to define abnormal blasts with an immunophenotype significantly different from the reference set in 7-dimensional space (light scatter and CD45 parameters excluded). C-Flow-MRD results were calculated by summating discrete abnormal blast populations above the limit of detection (LOD), and the assay result reported as the highest value of the two tubes of the AML MRD antibody panel. Analyses of specific progenitor compartments for C-Flow-MRD+ blast cells were performed in FlowJo software through progenitor pre-set sub-gating based on optimised thresholds.

### Statistical analyses

Receiver operating curve (ROC) statistics with area under the curve (AUC) were generated for MRD results of the clinical cohorts to summarise the discrimination ability of testing to predict relapse, with values of > 0.75 considered as good. Clinical specificity (true negative results (TN) ÷ [TN + false positives (FP)] x 100), sensitivity (true positive results (TP) ÷ [TP + false negatives (FN)] x 100), balanced accuracy ([%sensitivity + %specificity] ÷ 2) and Youden index ([sensitivity + specificity] – 1) were determined for test performance at specific assay cut-points. Optimal assay cut-points were derived from peaks in the Youden Index or alternatively by the R-based MaxStat package [16], which uses maximally selected rank statistics. Cumulative incidence of relapse (CIR) and treatment related mortality (TRM) from C-Flow-MRD applied to the previously published Figaro pre-transplant MRD sample dataset [14] were calculated using the ‘cumulative incidence of competing events and Gray test analysis’ and ‘Fine-Gray proportional hazard regression for competing events’ functions of the EZR software package v1.61 [17]. Further details are in Supplementary Methods.

## Results

### Flow cytometric MRD performance for detection of relapse during monitoring

To evaluate the diagnostic performance of routine flow cytometry MRD for detection of impending relapse at monitoring intervals of up to 4 months in a clinical setting, we interrogated the flow cytometric data of 136 MRD bone marrows preceding a paired relapse sample ( ≤ 4 months, median interval 2.45 months) from 118 patients who experienced relapse during longitudinal flow cytometric MRD monitoring (Supplementary Fig. 1, Table 1). MRD results were defined by MRD analysis that did not require a diagnostic sample (‘different-from-normal’/DfN approach, Methods).

**Table 1:** Characteristics by standard flow cytometric MRD status in pre-relapse samples.

	All *n* = 136	Flow MRD negative *n* = 47	Flow MRD positive		
			All *n* = 89	< 0.1% *n* = 17	≥ 0.1% *n* = 72
Interval between MRD sample and relapse Months, median [IQR]	2.45 [1.74–3.08]	2.77 [2.30–3.43]	2.27 [1.43–2.80]	2.80 [2.07–3.43]	2.12 [1.39–2.71]
Diagnostic Genetics					
Adverse cytogenetics or *TP53* mutated	46 (36%)	14 (34%)	32 (36%)	9 (53%)	23 (32%)
*FLT3* mutated	29 (22.5%)	13 (32%)	16 (18%)	4 (23.5%)	12 (17%)
MDS-related mutations	22 (17%)	6 (15%)	16 (18%)	1 (6%)	15 (21%)
Other	32 (25%)	8 (19.5%)	24 (27%)	3 (18%)	21 (30%)
*NPM1* mutated	5	1	4	4	0
*(FLT3* wild type)					
Unknown	7	6	1	0	1
Treatment Stage					
Post chemotherapy	48 (35%)	10 (21%)	38 (43%)	5 (29%)	33 (46%)
Post allograft	88 (64.7%)	37 (79%)	51 (57%)	12 (71%)	39 (54%)
Relapse major blast Immunophenotype					
CD34+	109 (80%)	44 (94%)	65 (73%)	12 (71%)	53 (74%)
CD34-CD117+	21 (15%)	3 (6%)	18 (20%)	5 (29%)	13 (18%)
Monocytic	0 (0%)	0 (0%)	0 (0%)	0 (0%)	0 (0%)
Other	6 (4%)	0 (0%)	6 (7%)	0 (0%)	6 (8%)

89 of these ‘clinical positive’ samples were MRD positive resulting in a diagnostic sensitivity for subsequent relapse in 2 to 4 months of 64% for a result of MRD ≥ 0.05% and 51% for MRD ≥ 0.1%.

We then assessed the specificity of a flow cytometric MRD test to predict relapse using two reference sets of MRD bone marrow samples from non-relapsing patients (clinical negatives). In the first set compromising 55 APML follow-up bone marrows (PCR negative, no subsequent relapse), 9% had MRD ≥ 0.05%. A similar frequency of clinical false positives (8%) was observed in the second reference set, consisting of 100 monitoring MRD bone marrows identified from higher risk AML patients with sustained off-treatment relapse-free survival > 6 months after the sample, (Supplementary Fig. 1, Supplementary Table 1). This second reference set of ‘clinical negatives’ was predominantly from transplanted patients with a comparable frequency of higher risk genetics at diagnosis (39% ELN 2O22 adverse, 41% *FLT3* mutations) to the relapse cohort (53% ELN 2O22 adverse, 23% *FLT3* mutations).

**Fig. 1:**
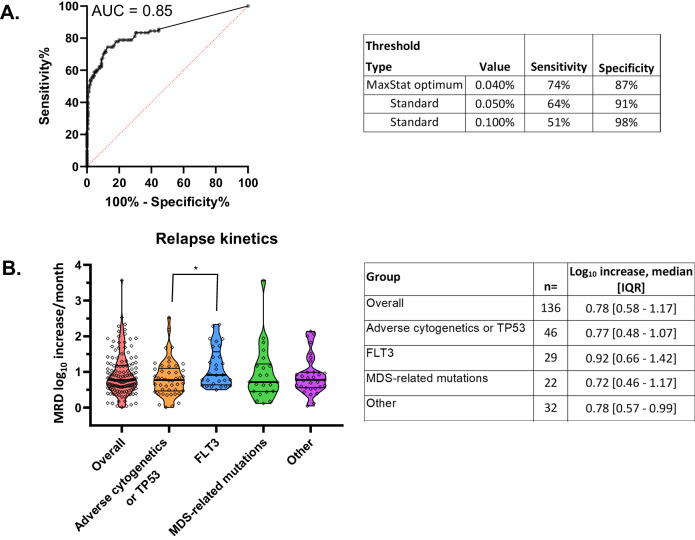
MRD relapse detection and kinetics of relapse by flow cytometric MRD monitoring. **A** Testing accuracy for pre-emptive detection of relapse by ‘different from normal’ MRD standard analysis. Receiver operating curve (ROC) statistics calculated from the combined cohort (136 true positives [relapse within 4 months of sample] and 121 true negatives [by sustained off-treatment remission after the sample]). Clinical specificity and sensitivity shown for routine assay thresholds (0.05% and ELN 0.10%) and optimum thresholds (derived from Maxstat package). **B** Relapse kinetics of high-risk AML. Relapse kinetics for 136 paired MRD and relapse bone marrows from 119 high risk AML patients. Relapse leukemic aberrant immunophenotypes were examined in MRD samples in parallel to standard ‘different-from-normal’ MRD analysis and kinetics calculated from the highest MRD values. Summary of relapse kinetics is shown for the overall sample cohort and specific diagnostic genetics subgroups; there was a significant difference (*P* = 0.026) in relapse kinetics between the *FLT3*-mutated and adverse cytogenetics /*TP53* subgroups by non-parametric Mann-Whitney U testing.

From the integrated dataset, MRD test specificity (for no relapse >6 months from test in the absence of intervention) was 91% for a result of ≥ 0.05%. From receiver operating characteristic curve analysis of combined pre-relapse and ‘clinical negative’ samples, the diagnostic accuracy of the routine flow cytometric MRD test to identify patients with imminent relapse ( < 4 months from test) was 0.85 (area under curve) with an optimal MRD clinical cut-off of 0.040% (sensitivity 74%, specificity 87%) in this retrospective cohort (Fig. 1A).

### Relapse kinetics

Kinetics of relapse can inform appropriate monitoring interval and choice of intervention but data as yet has been limited to PCR MRD targets. In this large sample cohort from 118 high risk AML patients experiencing relapse the median bone marrow increment was 0.78log_10_/month (IQR, 0.57-1.17), comparable to previous results for *NPM1* mutated AML [18]. We observed similar results between different high risk genetic subgroups although there was a significantly higher median increment in patients with *FLT3* mutations at diagnosis 0.92log_10_/month (IQR, 0.66–1.42) compared to patients with adverse risk cytogenetics or *TP53* mutations (*P* = 0.026) (Fig. 1B).

### Evaluation of MRD relapse detection using computational flow cytometric analysis

We developed a computational flow cytometric analysis tool incorporating a clustering algorithm and machine learning for standardised multidimensional MRD analysis (C-Flow-MRD) (Methods, Supplementary Methods). This performed different-from-normal’ unsupervised analysis against reference normal staging LPD BMs and required no baseline sample or expert annotation for MRD quantitation. The technical sensitivity of C-Flow-MRD from dilution experiments of different leukemic aberrant immunophenotypes (LAIPs) characterised by standard analysis was below 0.01% for most aberrancies (range 0.002- 0.02%) (Supplementary Fig. 2). The previously reported adverse prognostic impact of pretransplant MRD on relapse risk was recently confirmed in patients who entered the FIGARO trial, a randomized trial of reduced-intensity conditioning regimens [14]. The clinical validity of C-Flow-MRD was tested on this cohort of 156 transplanted patients. The prognostic performance of results generated by C-Flow-MRD was equivalent to routine Flow-MRD (Supplementary Fig. 3). We next applied C-Flow-MRD to the dataset of pre-relapse and all reference samples processed with the 8 colour AML MRD panel (Supplementary Methods). Optimal cut-off values by ROC analysis for detection of relapse were 0.036% and 0.08% with an equivalent clinical sensitivity to standard MRD (Supplementary Fig. 4.) There was good intertechnique agreement in MRD values between C-Flow and standard MRD (86% concordance, *r* = 0.83) (Supplementary Fig. 5A). Although some discrepant results were observed, including C-Flow-MRD+/standard MRD**-** values, these were reduced by the inclusion of myeloid control BMs into the reference set of control BMs (Supplementary Methods, Supplementary Fig. 5B).

### Hierarchy for relapse prediction by MRD is dependent on progenitor compartment

Depending on the AML genetic subtype, leukemic cells may be variably distributed between CD34+ and CD34- subpopulations, however data is lacking for how this impacts MRD detection. We examined this by C-Flow-MRD to enable extended, objective phenotypic characterisation of aberrancies between CD34+ and CD34- blasts. In our cohort of high-risk AML patients, clinical sensitivity for relapse by C-Flow-MRD was substantially from CD34+ aberrancies, however for 8 of 90 analysed pre-relapse samples, MRD was only detectable in the CD34-117+ compartment. CD34-CD117+ blast based MRD added up to 9% clinical sensitivity to CD34+ blast based MRD with MRD cut-offs ranging from 0.04% to 0.1% and had comparable clinical specificity (close to 90% when MRD was >0.08%). Notably only 3 of 8 patients with CD34-CD117+ restricted C-Flow-MRD had *NPM1* mutated AML (all 3 were also *FLT3*-ITD co-mutated). All relapse samples from these patients gained CD34+ aberrant populations, although CD34-CD117+ remained the predominant population (>95% of abnormal blasts) in 6.

**Fig. 2:**
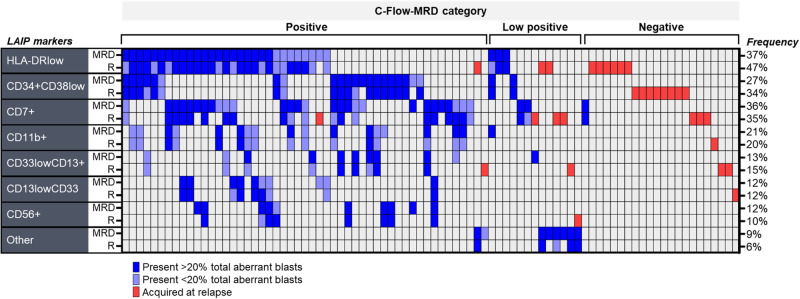
Aberrant phenotypes at MRD and Relapse. Each column represents one relapse. Samples grouped as MRD positive, MRD low positive and MRD negative by C-Flow MRD analysis of MRD timepoint. Results show presence for aberrant phenotype types detected at MRD timepoint (top 3 aberrancies of MRD blasts) and relapse timepoint (subsequent row, red = highest frequency aberrancy where top 3 MRD aberrancies are not detected).

Relapse-initiating cells may be enriched in certain hematopoietic stem and progenitor (HSPC) compartments associated with functional resistance through leukemic stem cell properties (CD34+CD38low) or immune evasion (such as HLADR downregulated HSPC). In our cohort that was skewed to post-allogeneic transplant relapses, C-Flow-MRD-detected aberrancies in the HLADRlow compartment provided the greatest effectiveness for clinical sensitivity and specificity (44% and 98% respectively above a cut-off of 0.02%) (Supplementary Fig. 6). Aberrancies of CD34+CD38low cells (LSC-type) contributed an additional 10% sensitivity (specificity of 93% above a cut-off of 0.02%) (Supplementary Fig. 6) notwithstanding the absence of certain LSC-specific markers (such as CD45RA, CLL-1 and CD123). We then examined incremental clinical sensitivity contributed by DfN aberrancies in other progenitor compartments that constitute established LAIPs using ELN core MRD markers. Of these, aberrancies in the CD7+ compartment provided the greatest increment in sensitivity for incipient relapse. Two thirds of pre-relapse samples were identified as C-Flow-MRD positive by one or more of the above three aberrancy categories whilst the cut-off of 0.02% maintained a specificity of ≥85% in the combined non-relapse samples (Supplementary Fig. 6). Most of the other tested aberrant subpopulations co-aggregated with these three (Fig. 2, Supplementary Fig. 7).

### Evolution in blast composition in months preceding relapse

**Fig. 3:**
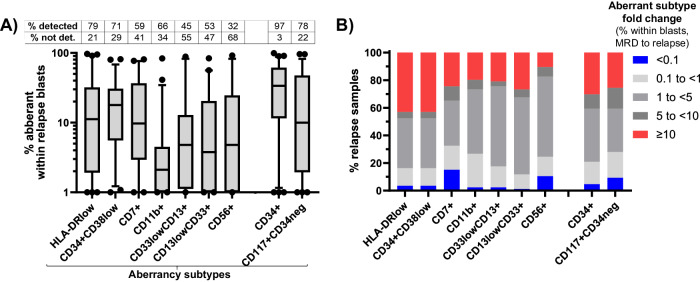
Relapse evolution of aberrant progenitor types within blasts. **A** Frequency of displayed aberrant phenotypic subtype within relapse sample blasts. Overall frequency for CD34+ and CD34- compartments shown at end of graph. Box plots indicate the median and interquartile range for relapse samples where aberrancy is detectable as assessed objectively by computational unsupervised analysis (whiskers = 5-95^th^ percentiles). Inset table displays detected versus non-detected frequency for each aberrant subtype (% of relapses). **B** Distribution of fold-changes in specific aberrant subtype frequency within blasts from pre-relapse MRD timepoint to relapse in paired samples (86 MRD samples paired with 76 relapses). Fold changes in total CD34+ and CD34- aberrancies shown at end of graph.

Non-genetic modes of evolution may potentially contribute to progression to relapse and could be reflected by changes in the abundance of specific immunophenotypic aberrant populations within the progenitor compartment. We investigated this by C-Flow-MRD in 86 paired pre-relapse (MRD timepoint) and relapse samples. In 94% of cases with pre-relapse MRD positivity, one or more of the 3 most frequent MRD aberrancies were present at relapse (Fig. 2). Consistent with previous observations [19, 20], LSC or HLADR-low type aberrant subpopulations were frequent (Fig. 2) and abundant in blasts across relapsed patients (Fig. 3A). Additionally, in over 40% of relapse samples with these populations, we observed rapid expansion (≥10 fold) within blasts during the pre-relapse interval (median 2.45 months) (Fig. 3B). Overall, rapid expansion in LSC and /or HLADR-low type aberrant blasts occurred for 59% (51/86) of paired MRD and relapse samples. Prevalence was similar across the higher risk genetic categories for HLADR-low subpopulations; however 60% of relapses in patients with MDS-like mutations had a rapid expansion of LSC-like subpopulations compared to 32% with *FLT3*-ITD mutated AML.

Other categories of detectable leukemic aberrant immunophenotypes had significantly lower abundance and relative expansion across patients. For example, CD11b+ type progenitor aberrancies, despite high MRD test specificity for relapse, remained as a relatively minor blast subpopulation (<10% of blasts) in most patients. We observed instability of certain aberrant populations between MRD and relapse, defined by a ≥ 10 fold reduction within blasts, in 21 of 86 relapse samples; 71% of the 21 had adverse cytogenetics and/or *FLT3* mutations. Instability was most frequently observed with CD7+ type aberrancies. In all cases of instability, 1 or more other detectable MRD aberrant blast subpopulations expanded within relapse blasts (examples provided Supplementary Fig. 8).

Evolution to relapse resulted in loss of the major (first ranked) aberrancy of MRD positive samples or a shift in its immunophenotypic profile (up to ~0.5 log decade change for 1 or more markers) in 20% and 2% of relapse samples respectively (Supplementary Fig. 9). Loss or shift was more frequent if MRD sample had only low level positivity. In ~50% of cases the first ranked MRD aberrancy was no longer predominant at relapse.

## Discussion

Although there is a clinical need for pre-emptive relapse detection in high-risk AML patients, there has been a gap in evidence for the predictive capability of serial off-treatment flow cytometric MRD testing. To address this, we evaluated flow cytometric MRD assay clinical performance in a large set of pre-relapse and non-relapse AML MRD samples. This evaluation was then extended to a potential tool for standardised interpretation of flow cytometric MRD, computational ‘different-from-normal’ flow cytometric MRD by an unsupervised analysis pipeline (C-Flow-MRD). Finally we applied the objective approach of C-Flow MRD to construct a hierarchy of aberrant progenitor compartments according to clinical specificity/sensitivity for relapse and examine their evolution profile in paired relapse samples.

Our results point to standard flow cytometric MRD as a highly specific predictor of relapse (98% ≥ 0.1%, 92% ≥ 0.05%) in higher risk AML for monitoring off-treatment by ‘different-from-normal’ analysis. This may obviate the requirement for a confirmatory repeat testing of a positive test as recommended based on experience from monitoring by PCR. Although over 60% of incipient relapses were detected by flow cytometric MRD ≥ 0.05%, this reduced to 50% when the higher current ELN threshold of 0.1% was applied. It is likely that extending monitoring interval beyond 2–3 months will further reduce clinical sensitivity. Our data underlines why a single MRD negative test should be interpreted with caution, notwithstanding the relatively short intervals of serial testing, due to rapid kinetics in a proportion of patients. The median log increase estimate per month in this high-risk cohort is comparable to that reported for *NPM1* mutated patients by PCR derived results (0.7 log_10_ per month) [18]. Consistent with clinical observations, results here show the highest median monthly log increase was apparent in the *FLT3* mutated subgroup even compared to those with adverse cytogenetics. More sensitive targeted deep sequencing-based assays for *FLT3*-ITD mutated variants (threshold of 0.01-0.001%) have the technical capacity to allow earlier MRD detection, but as a counterpoint, instability of these mutations risks false negatives, and the assays currently have a higher cost and slower turnaround time than flow cytometric MRD.

Importantly this study demonstrates the potential of computational unsupervised pipelines for analysis of flow cytometric MRD, not only to facilitate standardised interpretation but also to objectively distinguish aberrant immunophenotypes that have the greatest accuracy for pre-emptive relapse detection. Incorporating myeloid controls into the reference or ‘training set’ allowed further exclusion of what we term candidate ‘aberrant immunophenotypes with indeterminate potential’ (AIP-IP). Such AIP-IPs may be associated with clonal hematopoiesis [21–23] but screening for this by NGS molecular profiling was not possible in these samples. We anticipate that single cell genotypic and immunophenotypic approaches are most likely to resolve whether specific AIP-IPs are restricted to cells with clonal mutations or represent epigenetic mechanisms [24, 25]. Once AIP-IPs are identified, in our pipeline they can be incorporated into the unsupervised training sample set, analogous to the process that informs expert manual MRD analysis.

Unsupervised analysis by C-Flow-MRD allowed broad characterisation of immunophenotypic aberrant blast subpopulations. Leveraging this for the paired MRD and relapse samples, we examined blast composition longitudinally through disease progression in order to identify aberrant HSPC subtypes most likely to escape disease control. We observed positive selection of aberrant HSPC subpopulations with weak/negative expression of CD38 (on CD34+) and/or HLADR– a finding in ~45% of this high-risk AML cohort by the metric of >10-fold relative expansion within the blast compartment in the months preceding relapse. MRD detection of these subpopulations also had the highest accuracy for relapse. These results require validation with larger data sets and there are other limitations to consider. Aberrant subpopulation evolution is likely to depend on preceding treatments; the majority of our paired MRD and relapse samples were post-transplant. Additionally, our assay had a restricted number of markers and was limited to blasts expressing CD34 and/or CD117. However extending this approach to further paired pre-relapse and relapse sample sets in AML and MDS may map immunophenotypic compartments enriched for leukemic cells with competitive advantage versus neutral evolution. This provides a potential tool for targeted multiomic profiling of relapse cell subpopulations to decipher the properties of fitness advantage and rapid relapse kinetics according to treatment. Our findings also have implications with regards future optimisation of recently proposed strategies to improve MRD detection depth by mutational screening on sorted HSPC populations [26, 27].

In summary this study supports the utility of flow cytometric MRD for pre-emptive relapse surveillance in high-risk AML and provides a platform for the application of unsupervised MRD analysis to standardise and enhance evaluation of emerging leukemia.

### Supplementary information

Supplementary Figures

Supplementary Methods and Tables

## Data Availability

All data generated and analyzed in this study are included in this published article and its supplementary materials and figures. Raw datasets are available on reasonable request to corresponding author.
