# Supplementary material for: Pre-emptive detection and evolution of relapse in acute myeloid leukemia by flow cytometric measurable residual disease surveillance
Source: Leukemia. 2024 Jun 18;38(8):1667–73. doi: 10.1038/s41375-024-02300-z (PMC11286513; doi:10.1038/s41375-024-02300-z)
Supplement: Supplementary file 1 — Supplementary Figures [file 41375_2024_2300_MOESM1_ESM.pdf]

Supplementary Figure Legends

Supplementary Figure 1. Sample schema

Supplementary Figure 2. *Assay Performance characteristics of C-Flow-MRD*

A. *C-Flow-MRD limit of detection by serial dilution of diagnostic AML samples with different leukemic aberrant immunophenotypes*.

Diagnostic samples (n=13) were spiked into LAIP-negative bone marrows to achieve a low LAIP frequency of ~10^{-3} (0.1%) by standard MRD analysis, then diluted further 2- and 10-fold. Points represent the median of triplicate analyses where result values met the criteria of a minimum of 20 abnormal events (absolute LOD). Limit of quantitation (where CV of triplicates was <30%), was 0.04%.

LAIP, leukemic aberrant immunophenotype; LOD, limit of detection; CV, coefficient of variation.

B. *Sensitivity of C-Flow-MRD measured as frequency of blast compartment*

AML blasts from 21 diagnostic samples were spiked *in silico* into normal blasts at frequencies of 10%, 20%, 30%, 40%, 50% and 100%. Sensitivity of C-Flow-MRD was lower when LAIPs constituted <20% of AML blasts in diagnostic sample (grey lines) compared to >20% (black lines).

C. *Linearity of C-Flow-MRD*

Good result linearity was observed (median R^2, 0.996 and 0.980 for wet and *in silico* dilutions respectively). Lower result linearity was observed for *in silico* dilutions when LAIPs constituted <20% of AML blasts in diagnostic sample (poorer LAIP coverage, grey symbols).

D-E. *Precision of C-Flow-MRD analysis*

D. *Inter-analysis Precision*. Randomly selected pre-relapse sample files (n=13) were analysed in triplicate by varying the seed number for the FlowSOM clustering analysis.

E. *Intra-assay Precision* Triplicate values were derived from wet dilution experiments (34 samples between the 2 AML MRD antibody tubes).

Triplicate values are displayed as mean, bars represent standard deviation.

Precision values were calculated as the running mean %CV of 5 consecutive samples (ordered by median result value) (lower panel, green line). The running mean of the frequency of blast cells predicted to be abnormal by C-Flow-MRD was also determined (lower panel, red line). Intra-assay precision values remained acceptable at all frequencies measured. However, inter-analysis precision of the unsupervised pipeline was reduced when frequency of abnormal blasts measured was ≤3% of the blast compartment- vertical blue line represents the MRD threshold at which the %CV running mean exceeds the 40% threshold.

Supplementary Figure 3. *Clinical validation of C-Flow-MRD*

Outcomes according to the previously published results from standard flow cytometric MRD (**A**) were compared to computational flow cytometric MRD (**B**) for pre-transplant MRD bone marrows of the FIGARO trial cohort.

Relapse and non-relapse mortality are shown. Cut-off for MRD positivity was 0.2% for standard analysis (previously validated prognostically most discriminative threshold in this transplant cohort) and equivalent threshold (established by linear regression) for computational flow cytometric MRD.

TRM, transplant-related mortality.

Supplementary Figure 4. *C-Flow-MRD performance* for pre-emptive detection of relapse
Receiver operating curve (ROC) statistics by C-Flow-MRD ('different-from-normal' unsupervised analysis against reference normal staging LPD BMs).
Receiver operating curve (ROC) statistics of C-Flow MRD results for **test** (**A**) (45 relapse within 4 months and 55 non-relapse [APML] MRD samples), **validation** ((**B**) (45 relapse within 4 months and 100 non-relapse [non-APML, sustained off-treatment remission >6 month after the sample] MRD samples) and **combined (C)** cohorts. Thresholds shown represent optimised threshold values derived from the test sample cohort only (0.036% and 0.082%, where the former value aligns with the Maxstat optimal test cohort threshold.
Legend. C-Flow-MRD, computational (unsupervised) flow cytometric measurable residual disease; LPD, lymphoproliferative disease; BM, bone marrow; APML, acute promyelocytic leukemia, AUC = Area under the curve.

Supplementary Figure 5. *Cross technique comparison between C-Flow-MRD and standard Flow-MRD measurements*
Concordance/Correlation between % MRD values obtained from standard and C-Flow MRD measurements plotted on scatterplots. Symbols represents measurements from each sample. Correlation coefficients (r) were calculated by Pearson's correlation test with significance level shown. Perfect correlation would not be expected between the results of standard and C-Flow-MRD analysis due to methodological differences, where C-Flow-MRD represents the sum of discrete abnormal populations while standard analysis reports the frequency of the most abundant abnormality only.

- ***A.*** Displayed results are from the combined pre-relapse and non-relapser cohort (8-colour panel only, n=245) with C-Flow MRD measurements using normal staging LPD BM as reference set for 'different-from-normal' unsupervised analysis). Correlation was lower in non-relapse MRD samples due to predominanace of MRD values below positivity thresholds.
- ***B.*** Concordance /Correlation including MRD low positives between standard and C-Flow MRD before (LPD only reference) and after inclusion of false positive APML controls in reference set (LPD+APML reference) for 'different-from-normal' unsupervised analysis .

n.d, not detectable.

Supplementary Figure 6 *Hierarchy of aberrant progenitor compartments for effectiveness in relapse prediction*
Specific aberrant progenitor compartments were ranked serially for diagnostic performance by Youden's index (YI: sensitivity+specificity-1) (further detail in Supplementary Methods). Panel shows sequential YI with optimal MRD threshold achieving >95% specificity for each aberrancy type, followed by sensitivity, specificity and overall performance (balanced accuracy/B accuracy) for relapse prediction achieved by serial combination. HLADRlow, followed by LSC and CD7+ type aberrancies (detected above a threshold of 0.02%) contributed most to assay clinical sensitivity.

Supplementary Figure 7. *Aberrant phenotype co-occurrence in pre-relapse MRD samples*

Frequencies shown are the % of samples positive by phenotype (row heading) also positive by phenotype (column heading), or positive by no other phenotype ('Only'). Positivity is defined by C-Flow-MRD detection above threshold of 0.02% (0.01% for CD11b+) within these tested phenotypic compartments of blasts (CD34+ and/or CD117+). Applied gates and fluorescent intensity thresholds are shown in supplementary methods.

Legend. C-Flow-MRD, computational (unsupervised) flow cytometric measurable residual disease.

Supplementary Figure 8 *Aberrant immunophenotype profiles in patient paired MRD and Relapse samples with CD7+ MRD subpopulation instability*

Supplementary Figure 9. *Frequency of immunophenotypic shifts between MRD and relapse samples of first ranked (major) aberrancy in MRD sample.*

Aberrant immunophenotypes detected by C-Flow MRD were identical if in the same node(s) from higher dimensional unsupervised clustering (FlowSOM) and had minor immunophenotypic shifts (up to ~0.5 decade shift in fluorescent intensity of one or more parameters) if in the same metacluster but not the same nodes. Percentages on x axis are of all assessed relapse samples with a pre-relapse MRD positive (n=51) or low positive result (n=13). Results grouped by MRD sample result (positive vs low positive).

Supplementary Figure 10. *Gating Thresholds for CD38low and HLADRlow blast compartments*

A) Representative dot plot displaying CD34+CD38low/negative blast thresholds. The chosen optimal fluorescent intensity (FI) cut-off for CD38-FITC (FI=500) is shown, alongside a more stringent cut-off for CD38negative blasts (FI=200).

B) Representative dot plot displaying HLADRlow/negative blast threshold. The chosen optimal FI cut-off for HLA-DR-FITC (FI=800) is shown, alongside a more conservative cut-off (FI=300).

The selected thresholds (orange) performed with similar high clinical specificity, but improved clinical sensitivity, for patient relapse than the more conservative thresholds set at lower FI values.

Supplementary Figure 1. Sample schema

TRUE CLINICAL POSITIVES
for flow cytometric MRD monitoring interval of <4 months

Search criteria April 2015 - Sep 2022
- AML relapse samples with preceding [within 4 months] adequate MRD BM

136 MRD samples preceding 119 relapse samples (118 patients*)
- 107 patients* with 1x MRD sample within 4 months of relapse
- 13 patients with 2x MRD samples within 4 months of relapse
- 1 patient with 3 x MRD samples within 4 months of relapse

* Includes 1 patient with 2 relapses (22 months interval)

Fig 1 | Table 1

- 65 post HSCT
- 36% adverse cytogenetics / *TP53*
- 17% MDS-related mutations
- 23% *FLT3* mutations

C-Flow MRD performed on 90 MRD samples*
- 45 MRD samples (40 patients)in C-Flow Test cohort
- 45 MRD samples (36 patients in C-Flow Validation cohort

Fig 2 & 3

Fig S3

C-Flow MRD performed on 86 relapse paired with MRD samples**

Fig 4

*8 colour panel, excludes 46 MRD samples by 7 colour
**8 colour panel, excludes 4 pairs with non-identical fluorochrome

\

Supplementary Figure 1. Sample schema

TRUE CLINICAL NEGATIVES
for flow cytometric MRD monitoring interval of <4 months

Search criteria

Cohort 1 APML Post-treatment MRD BMs
October 2019-November 2021
Adequate sample
- PCR negative
- Confirmed molecular remission maintained at least 6 months after sample
- Consecutive BMs meeting criteria

→ N=55

Cohort 2 Higher Risk Off treatment MRD BMs
Dec 2020-January 2023
- Adequate sample
- MRD monitoring by ≥ 2 BMs
- Clinical information available
- Not ELN 2022 favorable risk if post chemo FU
- No subsequent intervention (including DLI)
- Confirmed continuous remission/RFS at least 6 months after sample
- BM PCR negative (if applicable)
- Consecutive BMs meeting criteria but prioritise consecutive BMs from same patient

→ N=100
- 99 post HSCT.
- 21% adverse cytogenetics / *TP53*
- 18% MDS-related mutations
- 41% *FLT3* mutations

\

Supplementary Figure 2. C-Flow-MRD analysis performance characteristics

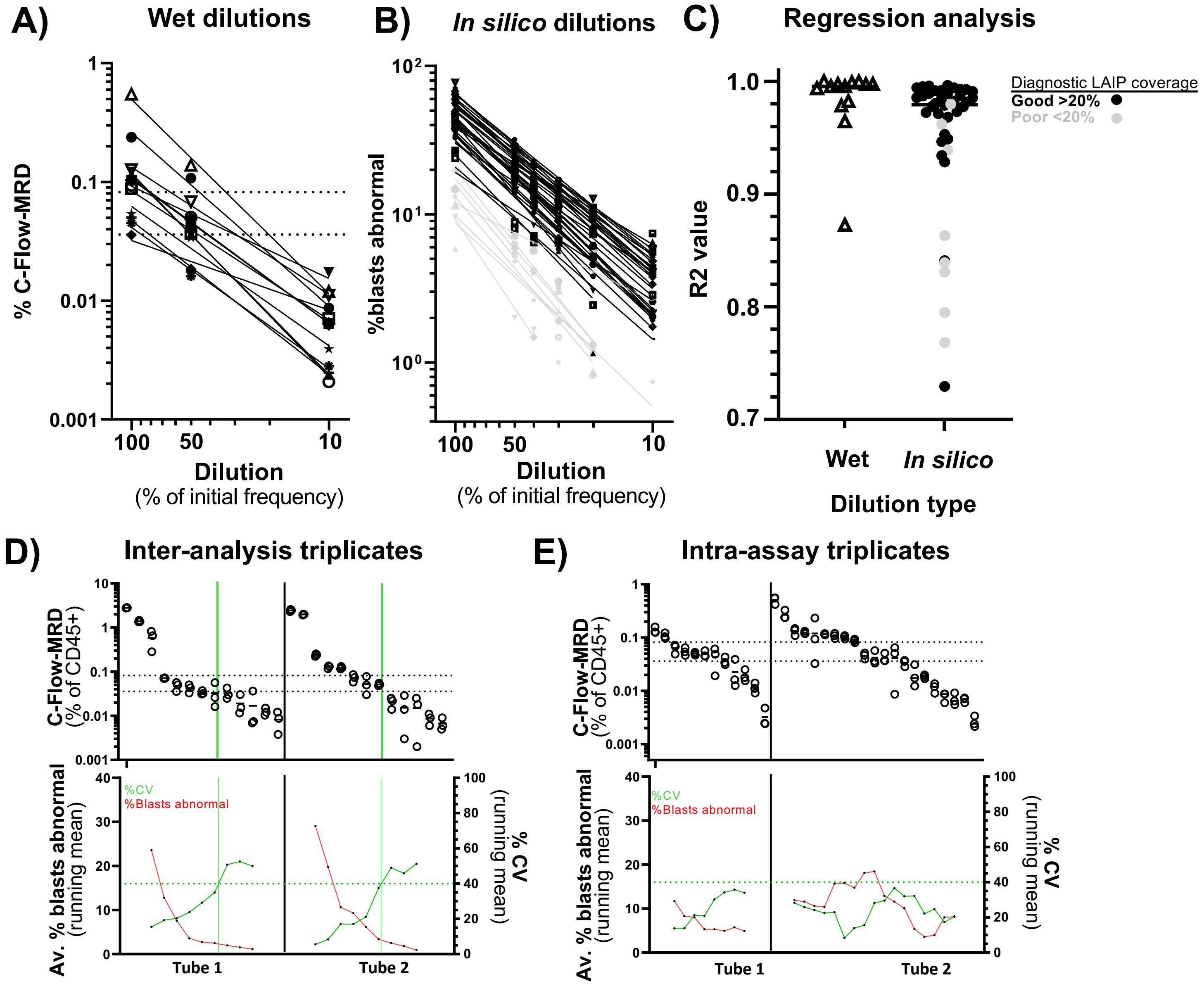

Supplementary Figure 3. Clinical Validation of C-Flow-MRD analysis

A. **pre-SCT MRD – standard MRD analysis**

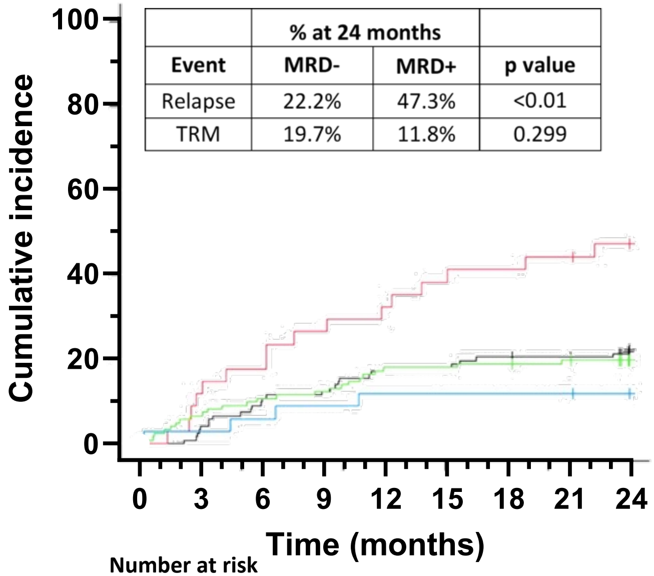

Number at risk

Negative	122	109	97	92	78	78	73	71
Positive	34	29	26	22	19	17	16	14

B. **pre-SCT MRD – C-Flow-MRD analysis**

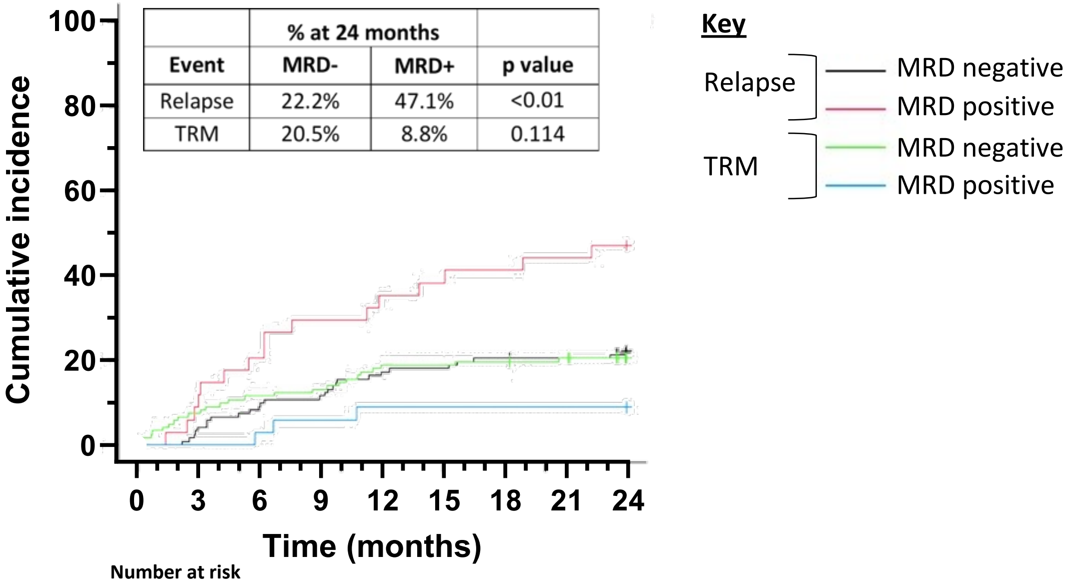

Number at risk

Negative	122	108	97	92	78	77	72	69
Positive	34	30	26	22	19	18	17	16

Supplementary Figure 4. C-Flow-MRD performance for pre-emptive detection of relapse

A. Test cohort ROC

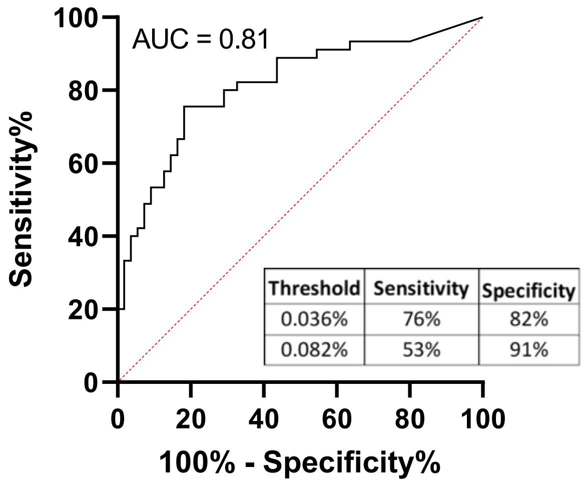

Threshold	Sensitivity	Specificity
0.036%	76%	82%
0.082%	53%	91%

B. Validation cohort ROC

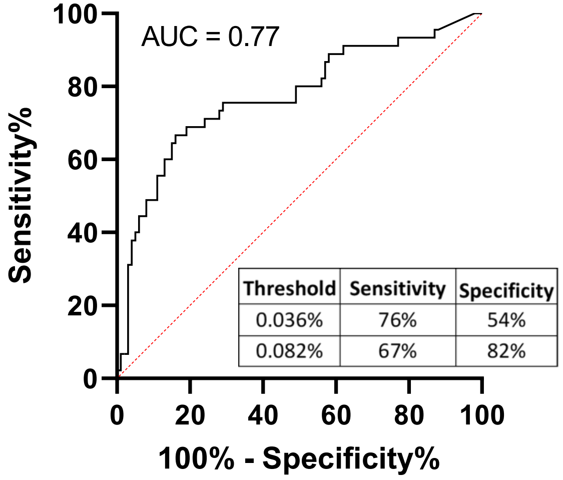

Threshold	Sensitivity	Specificity
0.036%	76%	54%
0.082%	67%	82%

C. Combined cohort ROC

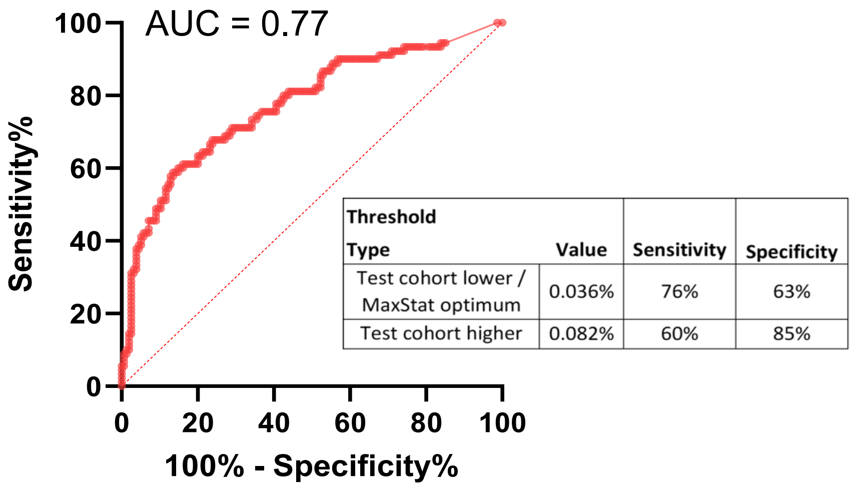

Threshold Type	Value	Sensitivity	Specificity
Test cohort lower / MaxStat optimum	0.036%	76%	63%
Test cohort higher	0.082%	60%	85%

Supplementary Figure 5.

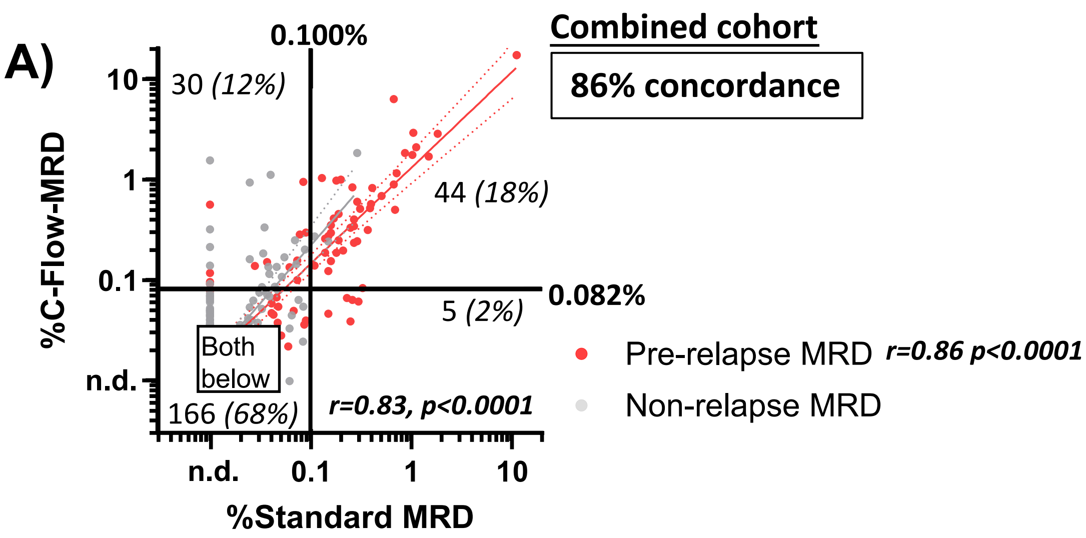

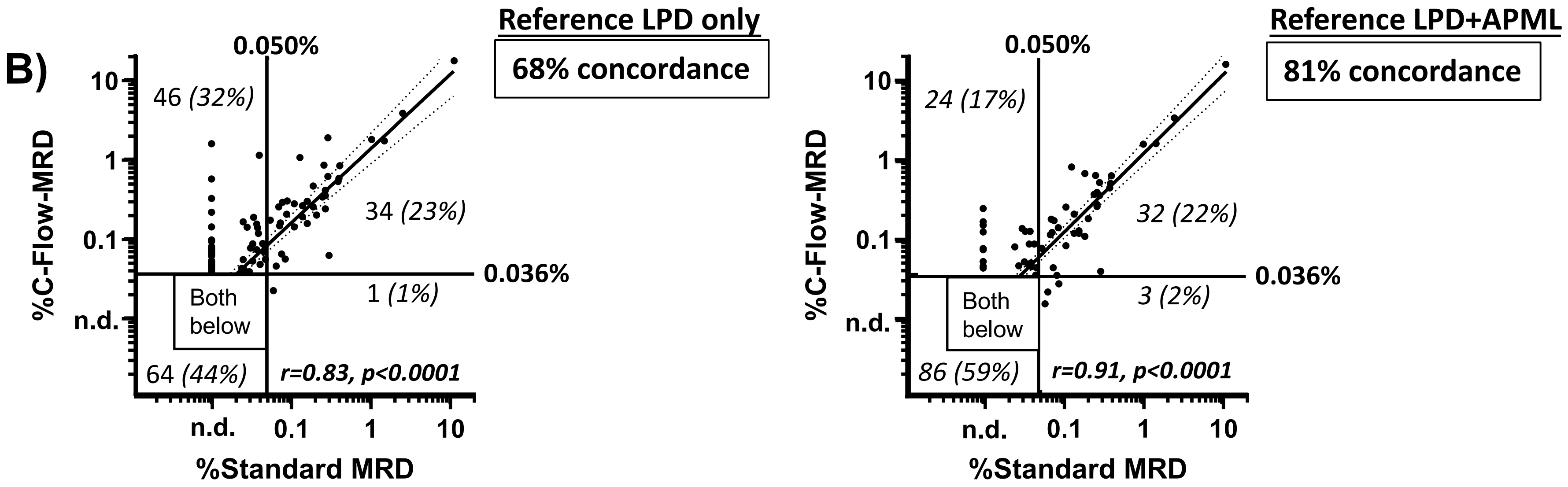

Supplementary Figure 6.

.

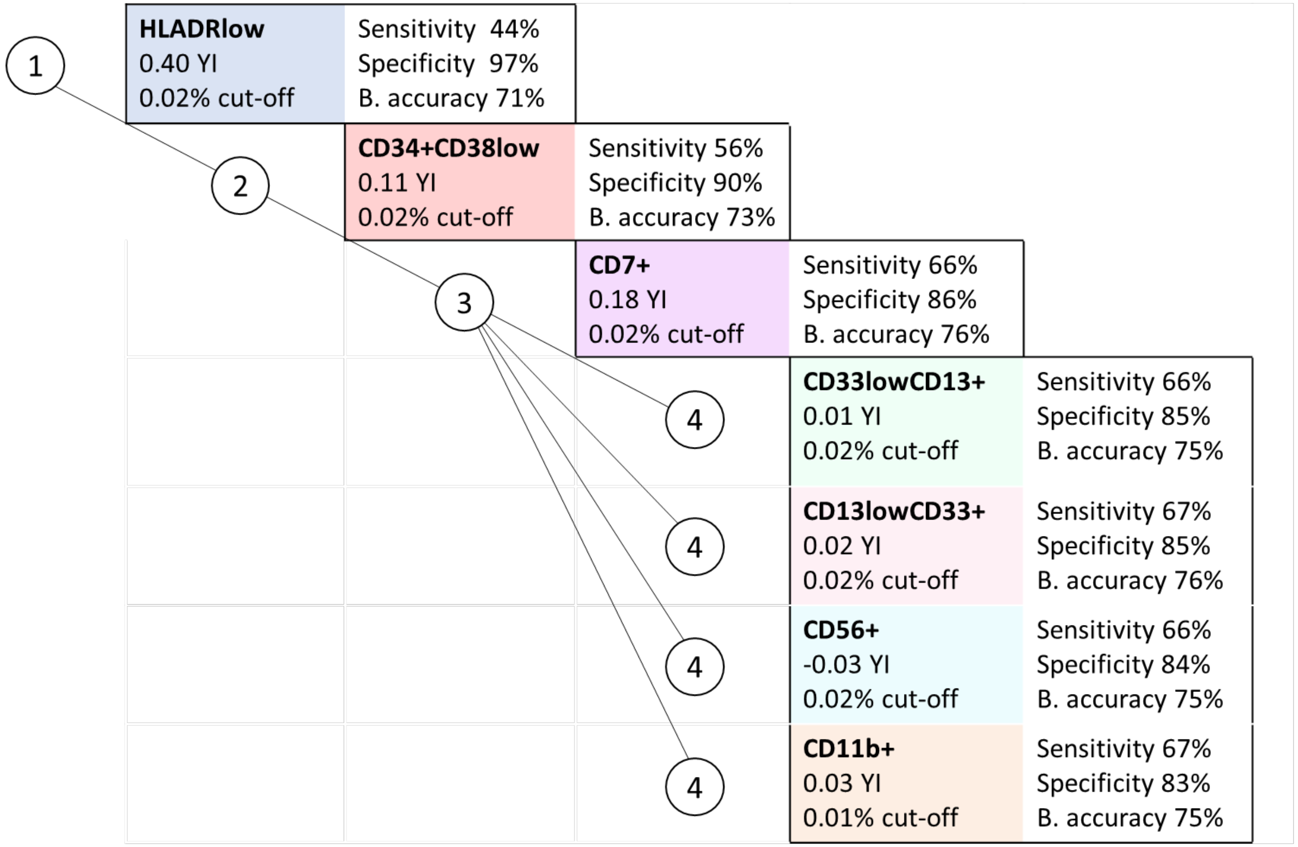

Supplementary Figure 7. Aberrant phenotype co-occurrence in pre-relapse MRD samples

	HLA-DRlow	CD34+ CD38low	CD7+	Any of previous	CD11b+	CD33lowCD13+	CD13lowCD33+	CD56+	Only
**HLA-DRlow**	100	58	53	-	65	35	38	30	5
**CD34+CD38low**	70	100	55	-	67	33	27	33	9
**CD7+**	60	51	100	-	57	29	23	31	17
**CD11b+**	76	65	59	97	100	32	35	35	3
**CD33lowCD13+**	88	69	63	94	69	100	19	25	6
**CD13lowCD33+**	94	56	50	100	75	19	100	50	0
**CD56+**	71	65	65	100	71	24	47	100	0

Supplementary Figure 8.

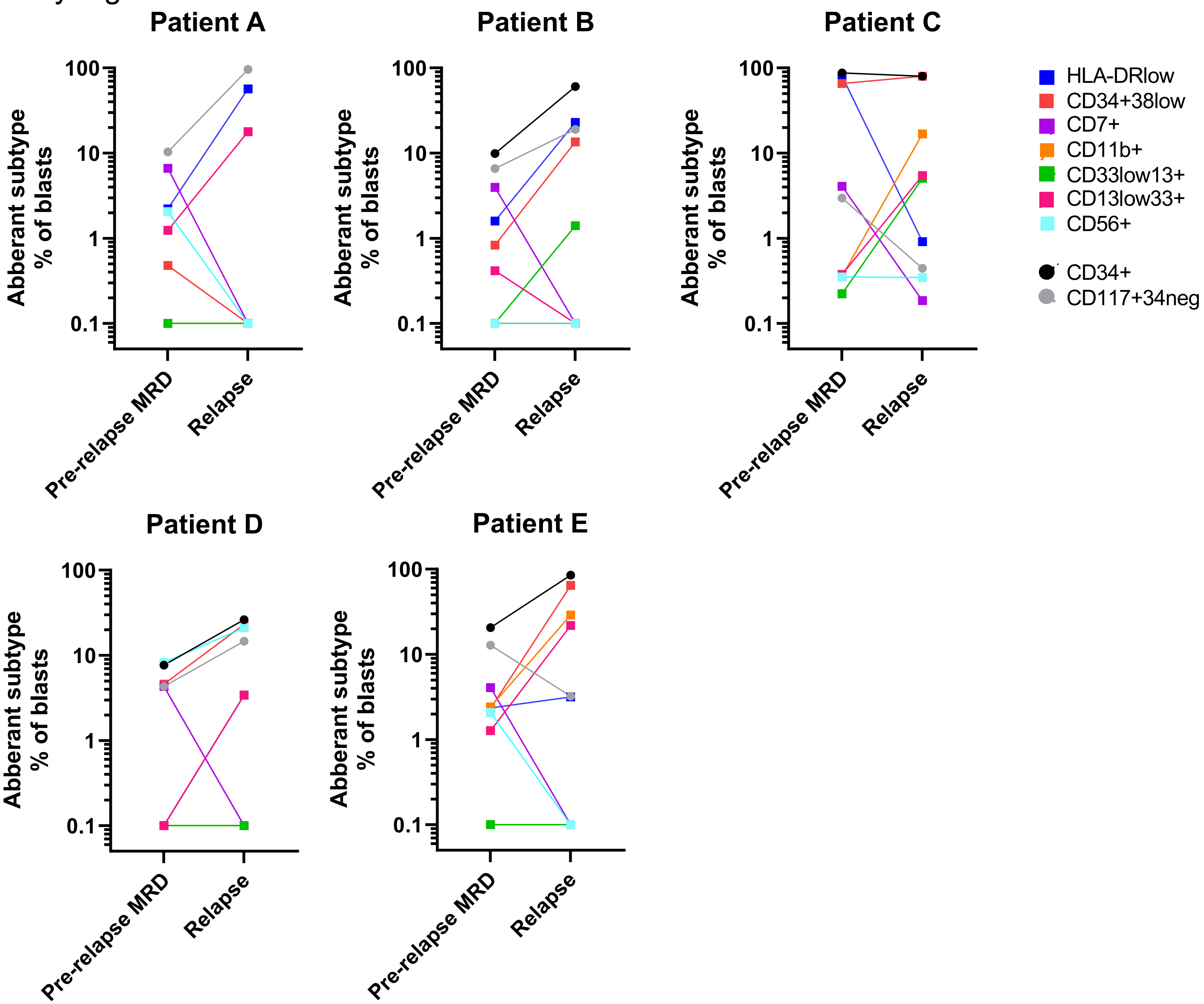

Supplementary Figure 9. Frequency of immunophenotypic shifts between MRD and relapse samples of first ranked (major) aberrancy in MRD sample.

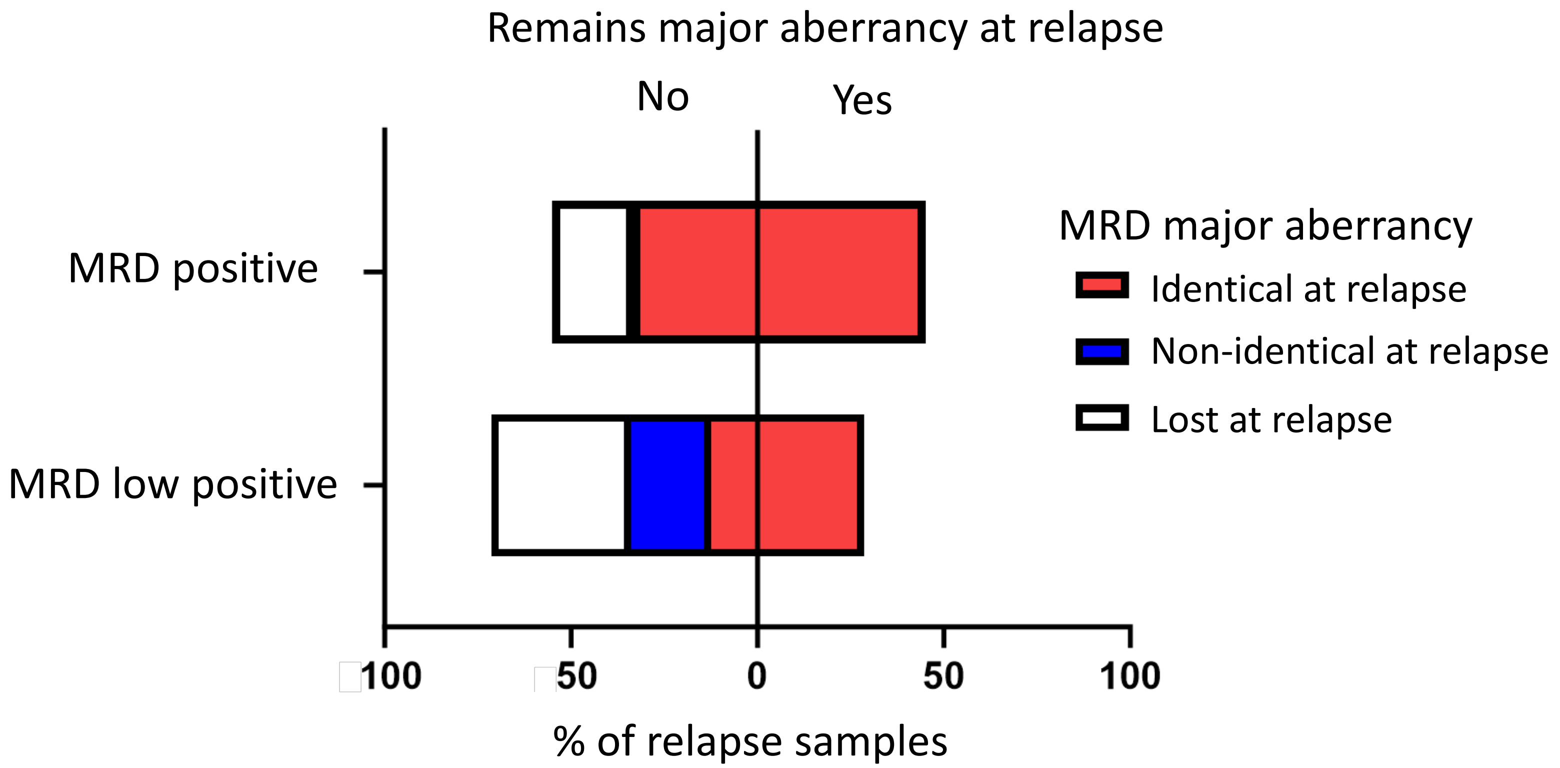

Supplementary Figure 10. Gating Thresholds for CD38low and HLADRlow blast compartments

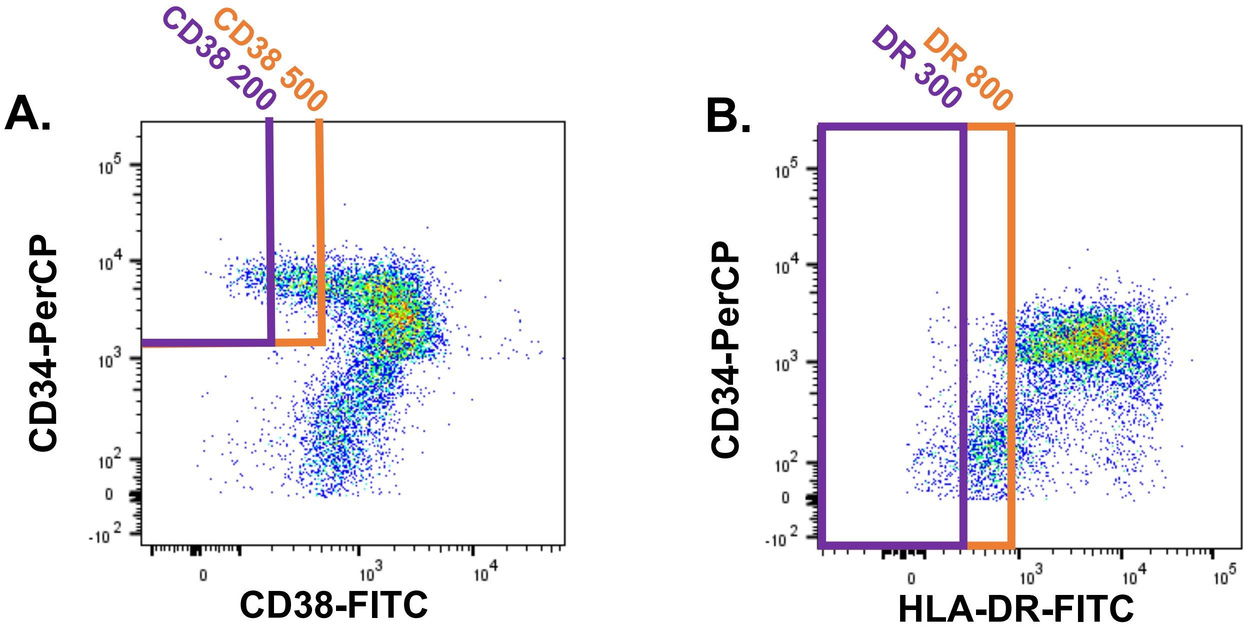

CD38neg threshold			
FI	Value	Sensitivity	Specificity
200	0.01%	18.8 %	97.5%
500	0.02%	32.2%	94.2%

HLA-DRneg threshold			
FI	Value	Sensitivity	Specificity
300	0.01%	31.1%	95.9%
800	0.02%	44.4%	96.7%
