# Supplementary material for: Pre-emptive detection and evolution of relapse in acute myeloid leukemia by flow cytometric measurable residual disease surveillance
Source: Leukemia. 2024 Jun 18;38(8):1667–73. doi: 10.1038/s41375-024-02300-z (PMC11286513; doi:10.1038/s41375-024-02300-z)
Supplement: Supplementary file 2 — Supplementary Methods and Tables [file 41375_2024_2300_MOESM2_ESM.pdf]

Supplementary Methods

Multiparameter Flow Cytometry (MFC) detection of MRD

Patients' samples were sent by overnight mail to the reference laboratory. Following ammonium chloride lysis, bone marrow nucleated cells were labelled with antibody panels shown in Supplementary Table 2A (7 colour tubes 1-3, applied until December 2017) and SupplementaryTable 2B (8 colour tubes 1-2 from December 2017) for flow cytometric MRD analysis also applied in UK NCRI AML trials as previously described.(1-3) Where performed, tube 3 of 7 colour panel provided information on myeloid maturation profile and an estimate of hemodilution (by CD11b/CD13 profile) but was not included in MRD analysis. Cell acquisition was performed on a FACSCanto (BD Biosciences) flow cytometer. Acquisition was set for 500,000 to 1 million cells or as many cell events as possible for MRD samples. Post-acquisition analysis of the flow cytometry data was performed (blinded to clinical data) using FlowJo software (Treestar Inc). Data review for analyses included periodically updated reference control bone marrow profiles. Viability, acquisition and autofluorescence artefact and hemodilution were assessed in acquisition generated flow cytometry standard (FCS) data files.

Standard (manual) flow cytometric MRD analysis

Routine MRD analysis in this cohort was performed using an analysis template with pre-set 'different-from-normal' regions (gates) applied to CD117+ and CD34+ blasts (gated by FSC/SSC/CD45/ CD117 or CD34), selected from previous data(2, 4, 5) on Leukemic Aberrant Immunophenotypes (LAIPs) that deviated from the normal antigen profiles with sufficient detection sensitivity (~0.02-0.05% sensitivity thresholds), usually comprised >10% of leukemic blasts at diagnosis and were known to be stable at follow-up. 'Different-from-normal' pre-set gates were 'empty' for control bone marrow CD117+ or CD34+ blasts (empty defined as $<10^{-4}$ mean+SD of >20 reference bone marrows). Screened 'different-from normal' pre-set gates are shown in Supplementary Table 3. Gates that included weak CD33 as a parameter were adjusted or excluded if myeloid CD33 expression was globally low. If 'different-from-normal' gates included events that might result from background (including artefact from autofluorescence), backgating was performed to check distribution of events in other marker and light scatter profiles. When there were increased myeloid blasts but no aberrant immunophenotypes from 'different-from-normal' regions, CD117+ and/or CD34+ leukemic blasts were overlayed with reference controls ('normal' CD117+ and/or CD34+ blasts) to further check for aberrant immunophenotypes. If there was an expanded myeloid blast population that was mainly or all negative for CD117 and CD34, blasts were gated by CD45/SSC or FSC/SSC then CD45intermediate and other markers (such as HLADR, CD56,

CD33, CD13) followed by overlaying with reference controls to identify LAIPs for which sensitivity threshold was at least 0.05% of leukocytes. Potential LAIPs that overlapped with mature monocyte profiles (usually because of higher CD45 expression) were not reported as MRD unless these predominated in patients with clear refractory disease by flow cytometry. Our panel was insufficiently comprehensive to discriminate monocytic LAIPs for MRD sensitivity.

MRD percentages were reported as percentage of leukocytes (CD45+) expressing the identified blast LAIP with the highest frequency and/or specificity and stability(6, 7). Any level of MRD detected above the sensitivity threshold for a baseline or previously defined LAIP was reported as MRD positive in FIGARO. MRD positivity was reported by 'different-from-normal' approach if >0.05% (or >0.1% when increased background below this level from autofluorescence / viability artefact) in the pre-set 'different-from-normal' gates. In some patients minor or major immunophenotypic changes from baseline or previous MRD sample LAIPs were detected but reported as MRD if fulfilled criteria for 'different-from-normal' approach. Inadequate follow-up samples defined by <0.1% blasts and/or <100 cell events within the total blast (gated by CD45/SSC plus CD34+ and/or CD117+ gate) were excluded from data analysis unless there was detectable MRD from a distinct aberrant cluster of at least 30-50 LAIP cell events.

Relapse Kinetics

Median increments per month were calculated from MRD values of pre-relapse MRD samples (samples <4 months from relapse) and paired relapse samples. When patients had more than 1 MRD sample (14 patients), kinetics of relapse datapoint represents average increment.

MRD values were assessed by different from normal (DfN) in CD34+ and/or CD117+ blast compartment (no major aberrant CD34-CD117- aberrant blasts detected at MRD timepoints). Relapse values obtained from predominat blast population including in CD34-CD117- compartment. Violin plots generated by Graphpad Prism are truncated at maximum and mimimum values.

Computational flow cytometric measurable residual disease analysis pipeline

Computational flow cytometric MRD (C-Flow-MRD) analysis consisted of four stages; pre-gating of myeloid blasts (FlowJo), cluster analysis (R-code), decision tree classification (R-code), and C-Flow-MRD result visualisation (FlowJo). Flow cytometry standard 3.0 (FCS3.0) list-mode data files were pre-gated in FlowJo version 10.7 software standardised template to export myeloid blast cells from each sample as a separate ".csv" file. Blasts were defined through exclusion of doublets, debris and data acquisition errors, followed by standardised

sequential gating on $CD45^+$ leukocytes, SSC^{low} mononuclear cells, and $CD117^+$ and/or $CD34^+$ cells, which were gated within the $CD45^{int}$ blast compartment. The control reference consisted of normal bone marrow control samples from patients under investigation for lymphoproliferative disease (LPD) with no significant aberrant population detectable in the marrow by flow cytometric analysis (LPD controls). Controls with global myeloid low CD33 expression were included. Each clustering run contained LPD control csv files (n=35-50) along with the MRD test file. Data were scaled to remove negative values (by random redistribution as a Gaussian variable for populations with negative values for relevant marker), and FlowSOM cluster analysis of each test sample run was limited to production of 225 nodes and 20 metaclusters. Decision tree analysis was applied to each metacluster in isolation to classify test sample blast populations of >20 events (absolute limit of detection, LOD) that exceeded a set probability threshold defining significant difference from the distribution of control cells in the decision tree terminal branches. Probability testing was performed in a pairwise manner with each individual LPD control file, and settings for both FlowSOM clustering and decision tree analysis were optimised for analytical sensitivity and specificity. Output FCS files were analysed in FlowJo software for the exclusion of auto-fluorescent background, and blasts labelled 'abnormal' by the classification parameter were quantified (%blast predicted abnormal). Finally, MRD results were calculated using the following formula; ["%CD117+/34+45int blasts in of $CD45^+$ events" x "%blasts predicted abnormal"] ÷ 100, and the sample C-Flow-MRD result was selected as the highest result value from AML tubes 1 and 2.

To assess the performance of C-Flow-MRD in reliably detecting residual leukaemic cells at low frequency, two sequential dilution datasets were produced. The first dataset consisted of laboratory dilution experiments, in which leukaemia associated immunophenotype (LAIP)-positive AML samples were diluted in LAIP-negative patient leukocytes to a target value of 5% LAIP coverage of $CD34/117^+45^{int}$ blast cells, and an initial target MRD value of 0.1% of CD45+ leukocytes (range 0.018-0.430%; IQR; 0.05-0.110%; median 0.078%), prior to further 2-fold and 10-fold serial dilutions. The second dataset included diagnostic AML samples spiked *in silico* at reducing frequencies (50%, 40%, 30%, 20%, 10%) into LPD control blasts. Linearity was determined for all samples in both dilution series by linear regression analysis (replicates excluded where C-Flow-MRD <20 event LOD). For the assessment of intra-analysis precision, samples were randomly selected from the pre-relapse MRD sample cohort for triplicate analysis by the C-Flow-MRD pipeline, in which the FlowSOM seed value was altered in replicate runs. Where determined, %CV values were calculated as follows – [standard deviation of triplicate samples ÷ sample mean] x 100 - and a running mean of %CV determined from 5 samples ordered sequentially by result value.

The C-Flow-MRD DfN assessment based on normal LPD staging BMs as reference controls was performed on the pre-relapse sample cohorts using two approaches; A) MRD sample alone and B) pre-relapse MRD sample with paired relapse samples in the same FlowSOM clustering run (to allow calculation of relapse kinetics, and immunophenotypic comparison of predicted abnormal blast populations between the MRD and relapse timepoint). C-Flow-MRD analysis results for pre-relapse MRD samples correlated very strongly (r2 = 0.97, slope 0.98) between analysis methodologies A and B.

Pre-relapse MRD samples were randomly and evenly allocated to either a 'test' cohort or 'validation' cohort (n=45 test/validation). The PCR-negative APML follow-up MRD samples (n=55) were assigned to the test cohort, and non-favourable risk, sustained remission patient MRD samples (n=66, genetic distribution shown in Supplementary Table 1) to the validation cohort. All samples in these cohorts were stained with the 8 colour MRD panel (Supplementary Table 2B).

Optimal cut-offs were informed by Youden Index (see *Methods: Statistics*) peaks in the test cohort, yielding C-Flow-MRD values of 0.036% (76% sensitivity, 82% specificity) and 0.082% (53% sensitivity, 91% specificity), which were subsequently applied to the validation and combined (test + validation) cohorts (Supplementary Figure 4). Only 1/211 combined cohort MRD samples had a 20 event LOD below this lower threshold (<56k total CD45+ leukocyte events), and C-Flow-MRD was positive above 0.082% for this sample, meaning all 211 samples had a sufficient technical LOD for result interpretation.

87% and 88% of C-Flow MRD results were concordant with standard Flow-MRD for the pre-transplant FIGARO and JANUS sample cohorts respectively.

The C-Flow-MRD training set was extended to include 10 APML control samples with outlier false positive results (detectable above 0.036%). We hypothesised that these false positive results represented candidate aberrant immunophenotypes with indeterminate potential. Excluding these for C-Flow-MRD testing on non-APML monitoring BMs resulted in fewer C-Flow-MRD/standard MRD discrepancies (Supplementary Figure 5B) and an improved specificity, that was most apparent at MRD low positive levels (74% vs 54% for MRD cut-off of 0.036%); comparable sensitivity for relapse was maintained.

To explore the relative contribution of progenitor compartments associated with functional resistance and /or known recurrent leukemia aberrant phenotypes, gates were optimised for the following blast compartments; HLA-DR$^{low/neg}$, CD38$^{low/neg}$(CD34^{+}), CD7^{+}, CD11b^{+}, CD33neg(13^{+}), CD13^{neg}(33^{+}) and CD56^{+}. Briefly, gates at varying fluorescence intensity (FI) values from 100-3k (markers outside of brackets only) were applied to predicted abnormal blast populations from all combined cohort samples. Optimal gating thresholds were chosen

where a gate-specific C-Flow-MRD cut-point in the range of 0.01-0.02% was established with clinical specificity of >95%, and maximal clinical sensitivity (thresholds displayed for HLA-DR$^{low/neg}$, CD38$^{low/neg}$(CD34^{+}) in representative dot plots of control blasts, Supplementary Figure 10). These gates were used to investigate MRD aberrant phenotype redundancy among pre-relapse MRD samples and calculate blast coverage of aberrant immunophenotypes for comparison between pre-relapse and relapse samples. In addition, Youden index was calculated for all potential cut-points achieving >90% specificity to inform a sequential gating model (test, validation and combined C-Flow MRD cohorts. The model was further checked for overfitting by repeated testing (x3) with random selection of 75% of both the relapse and non-relapse samples. This generated 1+2+3 gate results of 66/60/61% sensitivity, 88/87/87% specificity and 77/73/74% balanced accuracy.

Statistics

Unless otherwise specified, statistical testing and graph generation was completed in Graphpad Prism software (v10). Inter-group comparisons were performed using Mann-Whitney U tests (or Wilcoxin rank sum for paired analyses), linearity was assessed for dilution experiments and method result comparisons using linear regression analysis. Receiver operating curve (ROC) statistics with area under the curve (AUC) were generated for MRD results of the clinical cohorts to summarise the discrimination ability of the tests to predict relapse, with values of >0.75 considered as good. Clinical sensitivity, specificity, balanced accuracy and Youden index for a given assay cut-point were determined by the following formulae;

**Statistic**	**Formula**
Clinical specificity	true negative results (TN) ÷ [TN + false positives (FP)] x 100
Clinical sensitivity	true positive results (TP) ÷ [TP + false negatives (FN)] x 100
Balanced accuracy	[%sensitivity + %specificity] ÷ 2
Youden index	[sensitivity + specificity] - 1

Optimal assay cut-points were derived from peaks in the Youden index, or alternatively by the R-based MaxStat package(8), which uses maximally selected rank statistics. Cumulative incidence of relapse (CIR) and treatment related mortality (TRM) from the C-Flow MRD applied to the previously published Figaro pre-transplant MRD sample dataset(9) were calculated using the 'cumulative incidence of competing events and Gray test analysis' and 'Fine-Gray proportional hazard regression for competing events' functions of the EZR software package v1.61(10).

Supplementary Tables

Supplementary Table 1 Characteristics of clinical negative (non-relapse) sample cohort (All and by Standard and C-Flow MRD status)

	All Samples	Standard MRD negative	Standard MRD positive			C-Flow MRD positive*	
			All	<0.1%	≥0.1%	positive	low positive
	n=155	n= 111	n= 14	n= 11	n= 3	n=23	n=34
Cohort 1 APML<br>APML t(15;17)	55	50<br>(91%)	5<br>(9%)	4<br>(7%)	1<br>(2%)	5	5
Cohort 2 Higher Risk	100	91<br>(91%)	9<br>(9%)	7<br>(7%)	2<br>(2%)	18<br>(18%)	29<br>(29%)
Treatment stage<br>Post allograft<br>Post chemotherapy	 99<br>1						
Diagnostic Genetics							
Adverse cytogenetics or TP53 mutated	20<br>(21%)	20	0	0	0	2	5
FLT3 mutated	39<br>(41%)	36	3	2	1	6	9
MDS-related mutations	17<br>(18%)	12	5	4	1	6	6
Other<br><br>• *NPM1* mutated (*FLT3* wild type)	19<br>(20%)<br>2	18<br><br>2	1<br><br>0	1<br><br>0	0<br><br>0	4<br><br>1	6<br><br>0
Unknown	5	5	0	0	0	0	3

*C-Flow MRD performed by DfN with LPD control BMs as reference set.

Supplementary Table 2A AML- MRD 7-colour Antibody Panel

Tube No.	*FITC*	*PE*	*PerCP*	*PECy7*	*APC*	*APC H7*	*Horizon V450*
1	**HLADR**<br>*L243 (BD)*	**CD13**<br>*L138 (BD)*	**CD34**<br>*8G12 (BD)*	**CD117**<br>*1042D2 (BD)*	**CD33**<br>*P67.6 (BD)*	**CD45**<br>*2D1 (BD)*	**CD19**<br>*SJ25C1 (BD)*
2	**CD38**<br>*HB7 (BD)*	**CD56**<br>*MY31 (BD)*	**CD34**	**CD117**	**CD33**	**CD45**	**CD7**<br>*M-T701 (BD)*
3	**CD13**<br>*WM-47 (Dako, Alere)*	**CD11b**<br>*ICRF44 (BD Pharmingen)*	**HLADR**<br>*L243 (BD)*	**CD117**	**CD14**<br>MoP9 *(BD)*	**CD45**	

Supplementary Table 2B. AML- MRD 8-colour Antibody Panel

Tube No.	FITC	PE	PerCP	PECy7	APC	APC H7	BV 510	BV 421
1	**HLADR**<br>*L243 (BD)*	**CD13**<br>*L138 (BD)*	**CD34**<br>*8G12 (BD)*	**CD117**<br>*1042D2 (BD)*	**CD33**<br>*P67.6 (BD)*	**CD45**<br>*2D1 (BD)*	**CD14**<br>*SJ25C1 (BD)*	**CD11b**<br>*ICRF44 (BD)*
2	**CD38**<br>*HB7 (BD)*	**CD56**<br>*MY31 (BD)*	**CD34**	**CD117**	**CD33**	**CD45**	**CD19**<br>*(Biolegend)*	**CD7**<br>*M-T701 (BD)*

Supplementary Table 3. AML- MRD Standard analysis screened 'different-from-normal' aberrant immunophenotypes

'Different-from-normal' Aberrant Immunophenotypes<br><br>In CD34+ and /or CD117+ blasts
CD7+ (+/-CD33+)
CD56+ (+/-CD33+)
CD33wk/- CD13+
CD13 wk/- CD33+
HLADR wk/-
HLADR wk/- CD33+
CD34wk/- CD13intermediate CD117+
CD34wk/- HLADR+ CD117+
CD117- CD33+ CD34+
CD33+ CD38 wk/-
CD11b+
CD34high 117+
CD33high

Supplement References

1. Freeman SD, Craddock C. Selection of Conditioning Intensity for Allogeneic Hematopoietic Stem Cell Transplantation in Acute Myeloid Leukemia and Myelodysplasia - New Evidence Emerges. Transplant Cell Ther. 2021;27(6):443-5.
2. Freeman SD, Hills RK, Virgo P, Khan N, Couzens S, Dillon R, et al. Measurable Residual Disease at Induction Redefines Partial Response in Acute Myeloid Leukemia and Stratifies Outcomes in Patients at Standard Risk Without NPM1 Mutations. J Clin Oncol. 2018;36(15):1486-97.
3. Freeman SD, Thomas A, Thomas I, Hills RK, Vyas P, Gilkes AF, et al. Fractionated Versus Single Dose Gemtuzumab Ozogamicin with Determinants of Benefit in Older AML: UK NCRI AML18 Trial. Blood. 2023.
4. Freeman SD, Virgo P, Couzens S, Grimwade D, Russell N, Hills RK, et al. Prognostic relevance of treatment response measured by flow cytometric residual disease detection in older patients with acute myeloid leukemia. J Clin Oncol. 2013;31(32):4123-31.
5. Bradbury C, Houlton AE, Akiki S, Gregg R, Rindl M, Khan J, et al. Prognostic value of monitoring a candidate immunophenotypic leukaemic stem/progenitor cell population in patients allografted for acute myeloid leukaemia. Leukemia. 2015;29(4):988-91.
6. Schuurhuis GJ, Heuser M, Freeman S, Bene MC, Buccisano F, Cloos J, et al. Minimal/measurable residual disease in AML: a consensus document from the European LeukemiaNet MRD Working Party. Blood. 2018;131(12):1275-91.
7. Tettero JM, Freeman S, Buecklein V, Venditti A, Maurillo L, Kern W, et al. Technical Aspects of Flow Cytometry-based Measurable Residual Disease Quantification in Acute Myeloid Leukemia: Experience of the European LeukemiaNet MRD Working Party. Hemasphere. 2022;6(1):e676.
8. Hothorn T, Lausen B. On the exact distribution of maximally selected rank statistics. Computational Statistics & Data Analysis. 2003;43(2):121-37.
9. Craddock C, Jackson A, Loke J, Siddique S, Hodgkinson A, Mason J, et al. Augmented Reduced-Intensity Regimen Does Not Improve Postallogeneic Transplant Outcomes in Acute Myeloid Leukemia. J Clin Oncol. 2021;39(7):768-78.
10. Kanda Y. Investigation of the freely available easy-to-use software 'EZR' for medical statistics. Bone Marrow Transplant. 2013;48(3):452-8.
